# High-Content Phenotypic Profiling in Esophageal Adenocarcinoma Identifies Selectively Active Pharmacological Classes of Drugs for Repurposing and Chemical Starting Points for Novel Drug Discovery

**DOI:** 10.1177/2472555220917115

**Published:** 2020-05-22

**Authors:** Rebecca E. Hughes, Richard J. R. Elliott, Alison F. Munro, Ashraff Makda, J. Robert O’Neill, Ted Hupp, Neil O. Carragher

**Affiliations:** 1 MRC Institute of Genetics & Molecular Medicine, The University of Edinburgh, Western General Hospital, Edinburgh, UK; 2 Cambridge Oesophagogastric Centre, Cambridge University Hospitals NHS Foundation Trust, Cambridge, Cambridgeshire, UK

**Keywords:** esophageal adenocarcinoma, phenotypic, high content, mechanism of action, machine learning

## Abstract

Esophageal adenocarcinoma (EAC) is a highly heterogeneous disease, dominated by large-scale genomic rearrangements and copy number alterations. Such characteristics have hampered conventional target-directed drug discovery and personalized medicine strategies, contributing to poor outcomes for patients. We describe the application of a high-content Cell Painting assay to profile the phenotypic response of 19,555 compounds across a panel of six EAC cell lines and two tissue-matched control lines. We built an automated high-content image analysis pipeline to identify compounds that selectively modified the phenotype of EAC cell lines. We further trained a machine-learning model to predict the mechanism of action of EAC selective compounds using phenotypic fingerprints from a library of reference compounds. We identified a number of phenotypic clusters enriched with similar pharmacological classes, including methotrexate and three other antimetabolites that are highly selective for EAC cell lines. We further identify a small number of hits from our diverse chemical library that show potent and selective activity for EAC cell lines and that do not cluster with the reference library of compounds, indicating they may be selectively targeting novel esophageal cancer biology. Overall, our results demonstrate that our EAC phenotypic screening platform can identify existing pharmacologic classes and novel compounds with selective activity for EAC cell phenotypes.

## Introduction

Combined, the two major histological subtypes of esophageal adenocarcinoma (EAC) and esophageal squamous cell carcinoma represent the sixth leading cause of cancer deaths worldwide, with fewer than one in five patients surviving 5 y from diagnosis.^1^ A shift in epidemiology over the past 50 y has meant the incidence of EAC now vastly exceeds that of esophageal squamous cell carcinoma in Western countries,^2^ accounting for more than 80% of esophageal cancers in the United States.^3^ Defining the optimal neoadjuvant treatment regime is an area of active investigation,^4^ as current treatments carry a significant risk of systemic toxicity, histologic response rates remain poor,^5^ and only a limited subgroup of patients experience any survival benefit over surgery alone.^6,7^

EAC is a highly heterogeneous disease, dominated by large-scale genomic rearrangements and copy number alterations.^8^ This has made clinically meaningful subgroups and well-validated therapeutic targets difficult to define. Clinical trials with new molecular targeted agents have predominantly been directed toward epidermal growth factor receptor and human epidermal growth factor receptor 2 (HER2) receptors^9–12^ but thus far have proven unsuccessful. A potential explanation is the almost ubiquitous coamplification of alternative receptor tyrosine kinases and downstream pathways leading to redundancy and drug resistance.^8,13,14^ An alternative to target-based drug discovery, and increasing in popularity with technological advances, is phenotypic drug discovery, defined as the identification of novel compounds or other types of therapeutic agents with no prior knowledge of the drug target. Recent advances in phenotypic screening include automated high-content profiling.^15,16^ This approach involves quantifying a large number of morphological features from cell or small-model organism assays in an unbiased way to identify changes and phenotypes of interest. One benefit to this method is that a target does not need to be predefined, but the mechanism of action (MoA) of hit compounds can be inferred by reference to known compound sets using multivariate statistics and machine-learning approaches. Thus, this may prove a beneficial strategy for complex, heterogeneous diseases in which target biology is poorly understood and modern, target directed drug discovery strategies have made little impact on patient care, as exemplified by EAC.

Taking an unbiased, profiling approach to phenotypic screening, we chose to apply the Cell Painting assay to capture large amounts of information on cellular and subcellular morphology to quantify the cellular state across a panel of genetically distinct EAC cell lines. Cell Painting is an assay developed to capture as many biologically relevant morphological features in a single assay so as not to constrain discovery to what we think we already know.^17,18^ Therefore, upon chemical perturbation, we can detect changes in a subset of profiled features, allowing a phenotypic fingerprint to be assigned to a particular perturbation or compound.^15,19–21^ These fingerprints can then be used to identify specific phenotypic changes of interest, identify compounds that cause strong alterations in cell morphology suggesting changes in cellular state or stress, or predict MoA by similarity comparison to reference libraries of well-annotated compound mechanisms.^17,21^ However, this type of analysis is typically performed in a single “model” cell line, chosen for its suitability for image analysis. As a proof of principle that high-content phenotypic profiling could be applied to a panel of morphologically distinct EAC and tissue-matched control cell lines, we iteratively optimized cell culture conditions, cell-plating densities, and the Cell Painting assay staining protocol across our cell panel. Assay performance in terms of distinguishing distinct compound MoA for each cell type was evaluated by testing a small reference set of well-annotated compounds representing eight distinct mechanistic classes and performing principal component analysis (PCA) and t-distributed stochastic neighbor embedding (t-SNE) to visualize clustering of distinct mechanistic classes. We further developed a machine-learning model capable of predicting MoA across the panel of heterogeneous EAC cell lines. Following assay validation, we subsequently screened a library of 19,555 small molecules comprising target annotated probe compounds, approved drug libraries, and two diverse chemical sets with unknown MoA. PCA clustering of compound fingerprints distinguished a number of phenotypic clusters composed of similar pharmacologic classes active in the EAC cell lines. We also applied a Mahalanobis distance threshold and differential Z-score on our phenotypic data to identify compounds from our screen that were selectively active in EAC versus tissue-matched control cells. For prioritized hits, we have selected a subset and validated EAC selectivity with follow-up dose-response testing and performed transcriptomic pathway analysis pre- and posttreatment on sensitive and insensitive cell lines to further elucidate MoA. We further applied PCA and machine-learning analysis to phenotypic fingerprints from our diverse chemical set to identify compounds that exhibit selective activity on EAC cell phenotypes by a mechanism distinct from our reference set, indicating they may exhibit novel MoA.

Herein we describe the development and validation of a high-content phenotypic profiling assay and associated image informatics and machine-learning toolbox to classify the MoA of phenotypic screening hits across a panel of EAC and tissue-matched control cell lines. This approach has enabled the identification of chemical and target classes, including histone deacetylase (HDAC) inhibitors, which consistently cause the same cellular response across the panel of EAC lines, demonstrating efficacy against the heterogeneity of the disease. In addition, we identify pharmacologic classes such as the antimetabolites and new chemical entities with high selectivity for some EAC cell lines relative to tissue-matched controls. We propose that applying high-content multiparametric phenotypic profiling to a panel of genetically annotated EAC cell lines may stimulate new drug discovery and drug development programs for EAC through the identification of drug-repurposing opportunities and novel chemical starting points with selective activity for specific EAC genotypes.

## Materials and Methods

### Cell Culture

EPC2-hTERT cells were a kind donation from Anil Rustgis’s Lab, University of Pennsylvania.^22^

### Cell Line Authentication

Cell line identification (not carried out for the EPC2-hTERT line, as there is no reference sequence) was confirmed by short tandem repeat genotyping (Cell Line Authentication, Public Health England).

The cell lines were confirmed to be mycoplasma negative using the VenorGeM mycoplasma detection PCR kit (MP0025; Sigma, St. Louis, MO).

### Cell Subculture

EAC lines were grown in RPMI (#31870025, Life Technologies, Carlsbad, CA) supplemented with fetal bovine serum (10%) and L-glutamine (2 mM) and incubated under standard tissue culture conditions (37 °C and 5% CO_2_). The Barrett’s esophagus line (CP-A) and the esophageal epithelial line (EPC2-hTERT) were grown in KSFM (#17005075, Gibco, Carlsbad, CA) supplemented with human recombinant epidermal growth factor (5 g/L) and bovine pituitary extract (50 mg/L). Soybean trypsin inhibitor (250 mg/L, 5 mL) was used to neutralize trypsin.

### High-Content EAC Cell Painting Assay

Cells were seeded (50 µL per well) into 384-well, CELLSTAR Cell Culture Microplates (#781091, Greiner Bio-One, Kremsmünster, Austria) and incubated under standard tissue culture conditions for 24 h before the addition of compounds. CP-A cells were seeded at 800 cells per well, SK-GT-4 cells were seeded at 1000 cells per well, and the remaining cell lines were all seeded at 1500 cells per well.

Compound source plates were made at 1000-fold assay concentration and added to the cells with an overall dilution in media of 1:1000 from source to assay plate. Library concentrations are shown in **Supplementary Table S1**.

The primary screen was carried out as a single replicate, and the validation dose-response study was in triplicate.

After 48 h of incubation in the presence of the compounds, cells were fixed by the addition of an equal volume of formaldehyde (8%, 50 µL; #28908, Thermo Scientific, Waltham, MA) to the existing media, incubated at room temperature (20 min), and washed twice in phosphate-buffered saline (PBS). Cells were then permeabilized in Triton-X100 (0.1%, 50 µL) and incubated at room temperature (20 min) followed by two more washes with PBS.

The staining solution (**Table 1**) was prepared in bovine serum albumin solution (1%). Staining solution was added to each well (25 µL) and incubated in the dark at room temperature (30 min), followed by three washes with PBS and no final aspiration. Plates were foil sealed.

**Table 1.** Cell Painting Reagents, Concentrations, Excitation/Emission Wavelengths of the Filters Used for Imaging, and Suppliers.

Stain	Structure	Wavelength, ex/em (nm)	Channel	Concentration	Original Concentration^a^	Catalog No.; Supplier
Hoescht 33342	Nuclei	387/447	DAPI	4 µg/mL	5 µg/mL	H1399; Molecular Probes, Eugene, OR
SYTO 14	Nucleoli	531/593	CY3	3 µM	3 µM	S7576; Invitrogen, Carlsbad, CA
Phalloidin 594	F-actin	562/624	TxRED	0.14X	5 µL/mL	ab176757; Abcam, Cambridge, UK
Wheat germ agglutinin Alexa Fluor 594	Golgi and plasma membrane	562/624	TxRED	1 µg/mL	1.5 µg/mL	W11262; Invitrogen
Concanavalin A Alexa Fluor 488	Endoplasmic reticulum	462/520	FITC	20 µg/mL	100 µg/mL	C11252; Invitrogen
MitoTracker DeepRed	Mitochondria	628/692	CY5	600 nM	500 nM	M22426; Invitrogen

ex, excitation; em, emission.

a We also provide a comparison of reagent concentrations used in this study with the original Cell Painting protocol.^18^

### Image Acquisition

Plates were imaged on an ImageXpress micro XLS (Molecular Devices, Eugene, OR) equipped with a robotic plate loader (Scara4, PAA, UK). Four fields of view were captured per well using a 20× objective and five filters (**Table 1**). Each field of view typically contained 300 cells.

### Image Analysis

#### CellProfiler 2D image analysis

CellProfiler v3.0.0^23^ image analysis software was used to segment the cells and extract 733 features per cell per image. First, the pipeline identified the nuclei from the DAPI channel and used these as seeds to aid a segmentation algorithm to identify the cell boundaries from the TxRed channel, and finally these two masks were subtracted to provide the cytoplasm. These three masks marking the cellular boundaries were then used to measure morphological features including size, shape, texture, and intensity per object across the five image channels.

#### Image preprocessing

The cell-level data were aggregated to the image level by taking the median for each measured feature per image. Low-quality images and image artifacts were then identified and removed using image quality metrics extracted by CellProfiler. Images with fewer than 20 cells were also removed from the final analysis. For the remaining images, features were normalized on a plate-by-plate basis by dividing each feature by the median DMSO response for that feature. Features with NA values were removed, as were features with zero or near-zero variance, using the findCorrelation and nearZero functions in the R package Caret. All remaining features were scaled and centered globally by dividing by the standard deviation of each feature and subtracting the feature mean respectively. The pairwise correlations were calculated for all remaining features, and highly correlated features (>0.95) were removed. Finally, the image-level data were aggregated to the well (compound) level, and this was used in the analysis.

#### Random forest classifier

The random forest classifier was implemented using R’s Random Forest package with the following specified parameters: ntree = 500, data stratified by class, and sample size set to the smallest class size for balance. The images from three concentrations for each compound were pooled and treated as a single class. Two different analyses were run: first, MoA prediction was implemented for each cell line individually, and second, using leave-one-out cross-validation, one EAC cell line was left out of the training set at a time and that line was run as a test set.

PCA and t-SNE were implemented using the built-in R functions prcomp and RTSNE, respectively, to visualize the clustering of the compounds for each cell line.

#### Hierarchical clustering

Z-scores and Mahalanobis scores were centered and scaled for each compound across the panel of cell lines. Spearman correlation was then used to generate a distance matrix, and hierarchical clustering was determined using complete linkage.

#### NanoString transcriptomic analysis

Cells were seeded in six-well plates and incubated for 24 h. Media were then removed and replaced with DMSO (0.1%) or methotrexate (5 µM) in DMSO and incubated for 6 h. Cells were scraped and lysed using QIAshredders (#79654, Qiagen, Hilden, Germany), and RNA was extracted by means of the Qiagen RNeasy Mini kit (#74104, Qiagen; with β-mercaptoethanol) according to the manufacturer’s instructions and included a DNase digestion step (#79254, Qiagen).

Of the purified RNA, 100 ng was used as input for amplification-free RNA quantification by the NanoString nCounter Analysis System with the Human PanCancer Pathways and Metabolic Pathways panels. Raw counts were normalized to the internal positive controls and housekeeping genes using the nSolver 4.0 software.

## Results

### Assay Development

**Figure 1.**
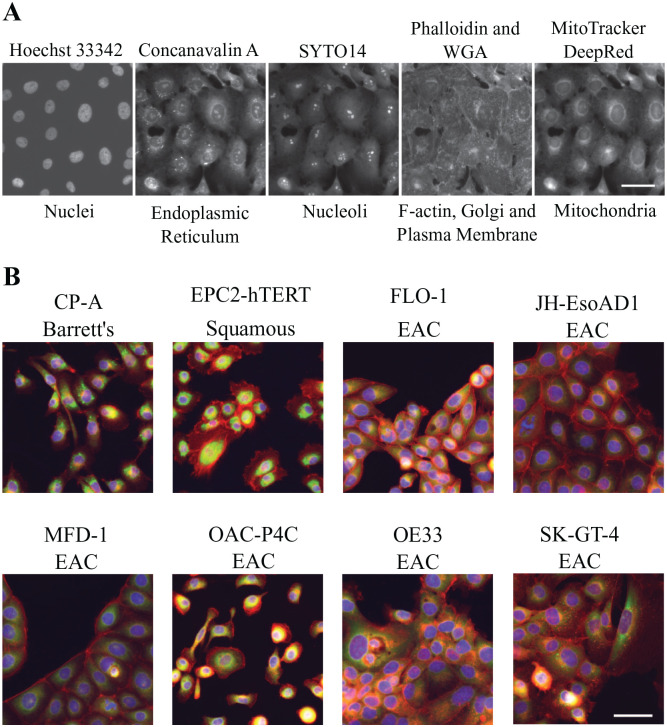
Cell Painting assay and the cell panel. (**A**) The five channels imaged in the Cell Painting assay for the representative cell line SK-GT-4, with dyes and cellular structures labeled. Scale bar is 50 µm. (**B**) Color combined representative control (DMSO) images of the eight cell lines in the cell panel: DAPI (blue), TxRED (red), FITC (green). Scale bar is 50 µm. See **Table 1** for additional details about the stains and channels imaged.

Because EAC is such a heterogeneous disease, we chose to develop a high-content phenotypic screening assay composed of a panel of EAC and tissue-matched nontransformed cell lines that captured this heterogeneity and thus provides a discovery platform for identification of novel targets and drug MoA that selectively target EAC. We assessed the amenability of 12 cell lines to high-content profiling, 10 EAC lines (JH-EsoAD1, FLO-1, MFD-1, OE33, OACM5.1, OAC-P4C, SK-GT-4, ESO51, ESO26, and OE19), and two tissue-matched nontransformed lines; a Barrett’s esophagus line CP-A, and a normal esophageal squamous line immortalized by expression of telomerase EPC2-hTERT. We assessed each cell line against a list of criteria that indicated high performance for high-content screening, including cell adhesion quality, cellular morphology, proliferation in 384-well plates, image segmentation, and MoA prediction accuracy. These criteria ensure image quality/information content, high-throughput screening compatibility, and image segmentation accuracy for downstream analysis pipelines. Based on suitable cell adhesion and morphological properties, we took forward the following eight cell lines for high-content assay development, including image segmentation and machine learning analysis: CP-A, EPC2-hTERT, FLO-1, JH-EsoAD1, MFD-1, OAC-P4C, OE33, and SK-GT-4 (**Fig. 1**).

The published Cell Painting protocol^17,18^ was adapted for our cell lines specifically as follows: the MitoTracker DeepRed was originally added before the cells were fixed; however, morphological changes have been seen in certain cell lines upon the addition of MitoTracker.^24^ Therefore, we opted to fix the cells first and add all of the Cell Painting reagents together after fixation to prevent artifactual morphological changes due to cell staining and to reduce complexity for robotic handling in a high-throughput setting. This also necessitated that we reoptimize the dye concentrations across our cell panel. Here, we increased the MitoTracker DeepRed concentration and reduced the concentration of Hoechst, Concanavalin A, and Wheat Germ Agglutinin and switched to a different phalloidin supply (**Table 1**).

### Machine Learning

Standard assay quality control metrics such as Z′Factor are unsuitable for multiparametric assays, particularly cell-based phenotypic profiling assays in which a desired phenotype is unknown and/or there is a lack of positive controls.^25–27^ To assess assay quality from a compound MoA profiling perspective, we used MoA prediction accuracy on a small well-annotated reference library of compounds with well-defined, known MoA (**Suppl. Table S2**). For this, we trained a random forest classifier using the CellProfiler extracted phenotypic information from the images of cells treated with the reference set of compounds.

Accuracy in the ability to predict MoA was used to assess whether the EAC and tissue-matched control cell lines were amenable to the phenotypic profiling assay, further validate whether image segmentation was accurate, and ensure that the phenotypic information extracted was relevant and broad enough to allow accurate prediction of MoA. To robustly evaluate compound selectivity and MoA across our heterogeneous panel of genetically distinct EAC cells, it was particularly important to assess the performance of each individual cell line and ensure that one cell line did not perform significantly better or worse than the others. A characteristic of EAC cell lines (OE33, MFD-1, and SK-GT-4 in particular) is the migration and formation of cell clumps, which are challenging to segment accurately by automated image analysis. Here we wanted to confirm that they were equal to the rest of the panel and suitable for the assay pipeline. OAC-P4C is a particularly morphologically heterogeneous line, so it was also important to ensure that image-level data can be used for phenotypic compound profiling in these types of cell lines.

**Figure 2.**
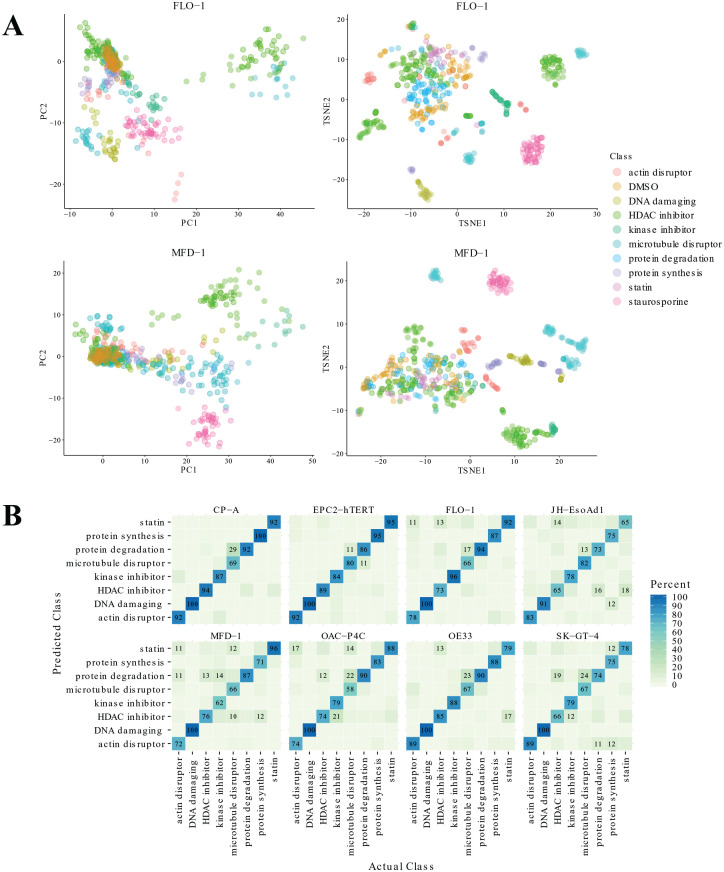
Reference library clustering and machine learning. (**A**) The first two components of principal component analysis (PCA) and t-distributed stochastic neighbor embedding (T-SNE) for the reference library compound treatments for the esophageal adenocarcinoma lines FLO-1 and MFD-1 (see **Suppl. Fig. S1** for remaining cell lines). Points are colored by mechanistic class, and multiple compounds concentrations are plotted. (**B**) Random forest classifier: confusion matrices of prediction accuracies per cell line in the cell panel for the reference library of compounds. Diagonal values show class sensitivities.

To visualize the phenotypic information extracted, we performed two data reduction methods, PCA and T-SNE, on the well-level data for the small reference library of compounds and plotted the first two components, colored by mechanistic class. PCA is a linear feature extraction technique, projecting the data in a lower-dimensional space while preserving the global structure of the higher-dimensional data.^28,29^ t-SNE is a nonlinear technique and is capable of capturing local as well as global structure.^30^ Input feature importance can also be examined when using PCA but not t-SNE. Given the complexity of the relationship between the features extracted from the images using both techniques allowed us to gauge feature importance, assess both a linear and nonlinear technique as there were no prior assumptions about the relationships between features, and look at both local and global trends in the data set. The results demonstrate that distinct compound classes generally cluster together. However, the strength of the phenotypic response varied across compound classes with some, such as the statins, producing a less distinct response than others. PCA clustering shows the statins are much closer to the DMSO controls (**Fig. 2**; **Suppl. Fig. S1**).

We next optimized a random forest classifier to test the MoA prediction on our reference library of well-annotated compounds. The extracted features from three concentrations of each compound were pooled and used to train the classifier. We chose 0.1, 1, and 10 µM, because using a broad range of concentrations means that each compound does not need to be optimized individually across each cell line.

When trained and tested on each individual cell line the average out-of-bag error^31,32^ was 20.38% across the entire panel of cell lines, ranging from 12% to 27%. The overall prediction accuracy for each cell line ranged from 73% to 88% across all eight compound classes, demonstrating the assay was well optimized across the panel. The weakest cell line was the SK-GT-4.

To confirm that the classifier was not overfitting, we used leave-one-out cross-validation.^33,34^ We implemented leave-one-cell-line-out and trained it on five of the EAC lines, testing on the remaining line. Here, as expected, it performed less well overall. However, the accuracy for each cell line ranged from 58% to 71% (**Suppl. Fig. S2**), indicating the ability of this classifier to be transferred to new cell lines despite having no prior training on them and thus the potential for the application of the classifier across a broader panel of cell lines without the need to train each cell line individually.

Overall, the accuracy of the machine learning demonstrates that the phenotypic profiling assay is of high quality across all eight cell lines, including morphologically heterogeneous cells, and feature extraction produces meaningful data for phenotypic analysis. The phenotypic profiling assay can therefore be applied to provide an initial evaluation of MoA of hit compounds influencing EAC cell proliferation, survival, and morphology. As such, our multiparametric high-content phenotypic profiling assay may prove useful in the prioritization of compound hits, which represent novel MoAs, and the deprioritization of compounds, which represent undesirable MoAs for subsequent medicinal chemistry and target deconvolution investments. We therefore prioritized the full panel of eight lines that passed our quality control criteria (six EAC lines with diverse genetic backgrounds, a Barrett’s esophagus line, and a nontransformed squamous esophageal line) for a phenotypic screen of 19,555 small molecules.

### Small-Molecule Screen

A total of 19,555 small molecules, including approved drugs, were profiled against our panel of eight cell lines using the ImageXpress microXL high-content imaging platform. Cells were treated with the commercially available Prestwick Chemical Library of 1280 mostly off-patent drugs, the LOPAC library of pharmacologically active compounds (1280 compounds), a proprietary diverse chemical library provided by CRUK Therapeutics Discovery Laboratories (Cambridge; 13,408 compounds), the BioAscent library of 3200 compounds, and bespoke libraries of 387 target-annotated compounds and chemical probes. The primary phenotypic screen across all eight cell lines encompassed 512 × 384 well plates, 3.9 million images, and 36 TB of data in total. Image analysis was performed using CellProfiler across a computer cluster.

Using a panel of cell lines better represents a heterogeneous disease and allowed us to identify compounds that demonstrated selective activity across multiple EAC lines and not in the tissue-matched control. We ran two parallel analyses for primary hit selection against the EAC lines: one based on broad, morphological, phenotypic changes and the other on cell growth and survival using nuclei count. At cytotoxic concentrations, there are few attached cells, and these are often rounded up, leading to a lack of information in the images. Therefore, images with 20 or fewer cells were removed from the morphological analysis.

We began our analysis with a subset of 3000 annotated compounds (excluding the CRUK Therapeutics Discovery Laboratories and BioAscent lead-like molecules).

To identify compounds inducing strong phenotypic changes, we used PCA on the feature data to reduce the dimensions and then calculated the Mahalanobis distance to the DMSO controls for the first 15 principal components, which explain approximately 90% of the variation in the data across each cell line. The Mahalanobis distance measures the distance of each point from the data distribution (in this case, the DMSO controls). The data distribution takes into account the mean and the spread of the data points using the covariance matrix as a normalization factor.^35^ It therefore addresses problems of both scale and correlation of the variables and is particularly beneficial for large multivariate data sets. This leads to elliptic rather than circular decision boundaries, as is the case for Euclidean. We therefore chose to use it as an unbiased metric of compound activity upon each cell line in the screen.

**Figure 3.**
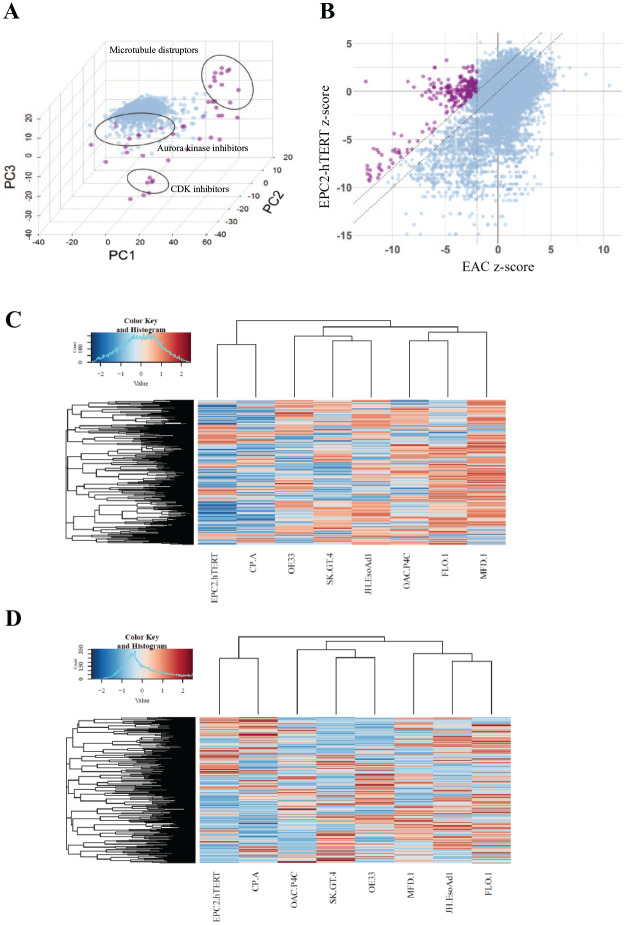
Hit analysis. (**A**) The first three components of principal component analysis (PCA) for exemplar data from the esophageal adenocarcinoma (EAC) cell line; JH-EsoAD1. Hits (purple) are defined as having a Mahalanobis distance of greater than 1500 from the DMSO controls. (**B**) Z-score plot for all EAC lines overlaid versus the EPC2-hTERT esophageal squamous control line. Hits (purple) are defined as having a z-score of −3 or greater in the EAC lines and showing selectivity of at least 2 z-scores compared with the EPC2-hTERT line. (**C**) Z-score hierarchical clustering of the cell panels’ response to compounds. (**D**) Mahalanobis distance clustering of phenotypic response to compound treatments across cell lines.

Phenotypic analysis identified 62 compounds that selectively target two or more of the EAC lines over the nontransformed esophageal cells. Clustering the cell panel’s responses to these molecules showed a number of phenotypic clusters enriched with similar pharmacologic classes, including HDAC inhibitors, microtubule disruptors, and antimetabolites, suggesting that hits have clustered mechanistically (**Fig. 3A**; **Suppl. Fig. S3**).

Based on cell growth and survival (i.e., nuclei count), we identified 27 compounds that were selectively active in two or more of our EAC lines. Here, hits were defined as having a z-score of −3 or greater in the EAC lines and a difference of at least 2 in one or both of the control cell lines (e.g., for a hit with a z-score of −3 in an EAC line, the z-score in the EPC-2 would have to be greater than or equal to -1). This comparison was made between each EAC line and the control lines to define hits and then selected if they were selectively active in at least two EAC lines across the panel (**Fig. 3B**).

Compounds from the growth and survival analysis cluster into several therapeutic classes, suggesting mechanistic pathways that may be selective for EAC cell growth and survival. Classes include antimetabolites and HDAC inhibitors. These classes were also identified in the morphometric phenotypic analysis (**Suppl. Fig. S3**).

We performed hierarchical clustering of cell-line responses to the compounds, as determined by the Mahalanobis metric (morphometric phenotypic analysis) and the z-scores (nuclei count; **Fig. 3C, D**), enabling pharmacologic discrimination of cell lines. These results show that the control cell lines, EPC2-hTERT and CP-A, can be discriminated from the EAC panel based on global drug screening data, providing confidence that our high-content Cell Painting assay can identify compounds with selectivity for EAC over the tissue-matched control lines.

### Antimetabolites Are Selectively Lethal to EAC Cells

From the subset of 3000 annotated compounds, we identified the drug methotrexate and three other structurally related antimetabolites, pemetrexed, raltitrexed, and aminopterin, as highly selective for EAC cell lines relative to tissue-matched control CP-A and EPC2-hTERT cells in both the nuclei count and morphological phenotypic analyses. We therefore validated this class of compound for dose-dependent activity. Aminopterin was removed from further analysis because of its toxicity profile in the clinic^36^; however, it showed potent activity in an initial dose response in the EAC lines, validating it as a hit from our screen (results not shown).

**Figure 4.**
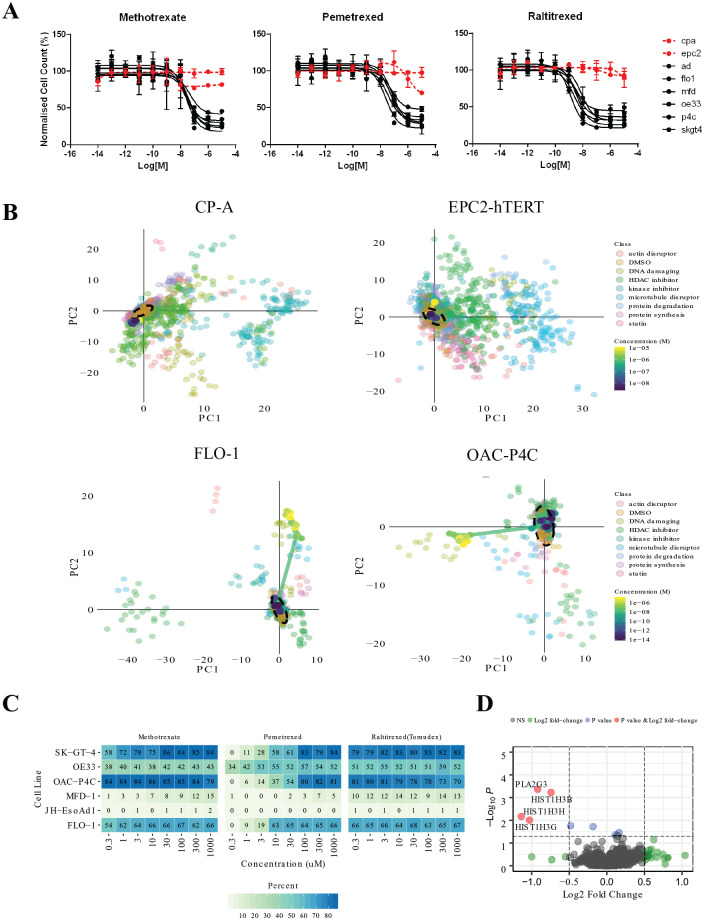
Antimetabolite evaluation. (**A**) Dose responses for methotrexate, pemetrexed, and raltitrexed across a panel of cell lines. (**B**) Principal component analysis of dose responses overlaid on the reference library for methotrexate in two resistant lines (CP-A and EPC2-hTERT) and two sensitive lines (FLO-1 and OAC-P4C). (**C**) Probabilities expressed as percentages for DNA damaging class for each cell line and each of methotrexate, pemetrexed, and raltitrexed. (**D**) Differential expression analysis for methotrexate treatment (5 µM, 6 h) for FlO-1, SK-GT-4, and OE33 cell lines. Red indicates genes reaching both the *p*-value and fold-change threshold, blue indicates genes that reached the *p*-value threshold, and green indicates genes that reached the fold-change threshold. *p* = 0.05, log_2_-fold change = 0.5.

Nuclei count dose responses for methotrexate, pemetrexed, and raltitrexed demonstrated strong selectivity against the EAC lines and showed minimal cytotoxic or phenotypic activity in either the CP-A or the EPC2-hTERT line even at 10 µM (**Fig. 4A**; **Suppl. Table S3**), validating our hit selection criteria.

Multiparametric phenotypic dose-response profiles of the antimetabolites overlaid on the reference library of annotated compounds (**Suppl. Table S2**) showed strong dose-dependent phenotypic changes, moving from phenotypically inactive (clustering with DMSO controls) to clustering with the DNA-damaging agents at active concentrations (**Fig. 4B**; **Suppl. Fig S4**) in all but the JH-EsoAD1 and MFD-1 lines. All three compounds also showed little or no effect in the control lines EPC2-hTERT and CP-A, clustering closely with the DMSO controls at all concentrations tested.

Class probabilities from the pretrained machine-learning model for each of the compounds predicted that they belong to the DNA damage class for all but the MFD-1 and JH-EsoAD1 lines (**Fig. 4C**), consistent with the clustering above. Probabilities also increased in a dose-dependent manner, indicating that cellular phenotypic activity follows a linear on-target dose-response relationship. These results further confirm the ability of the Cell Painting assay to accurately predict the MoA of validated hit compounds.

NanoString differential expression analysis^37^ revealed methotrexate treatment caused a significant reduction in the expression of Histone H3 subunits (HIST1H3B, HIST1H3G, HISTH3H; **Fig. 4D**) in the sensitive cell lines only, with no effect in either of the tissue-matched controls (**Suppl. Table S4**). Several other genes changed with methotrexate treatment, but none were significant. Further mechanistic studies are required to further elucidate how and if such expression changes confer selectivity to methotrexate.

### Toward Novel Therapies and Targets for EAC

**Figure 5.**
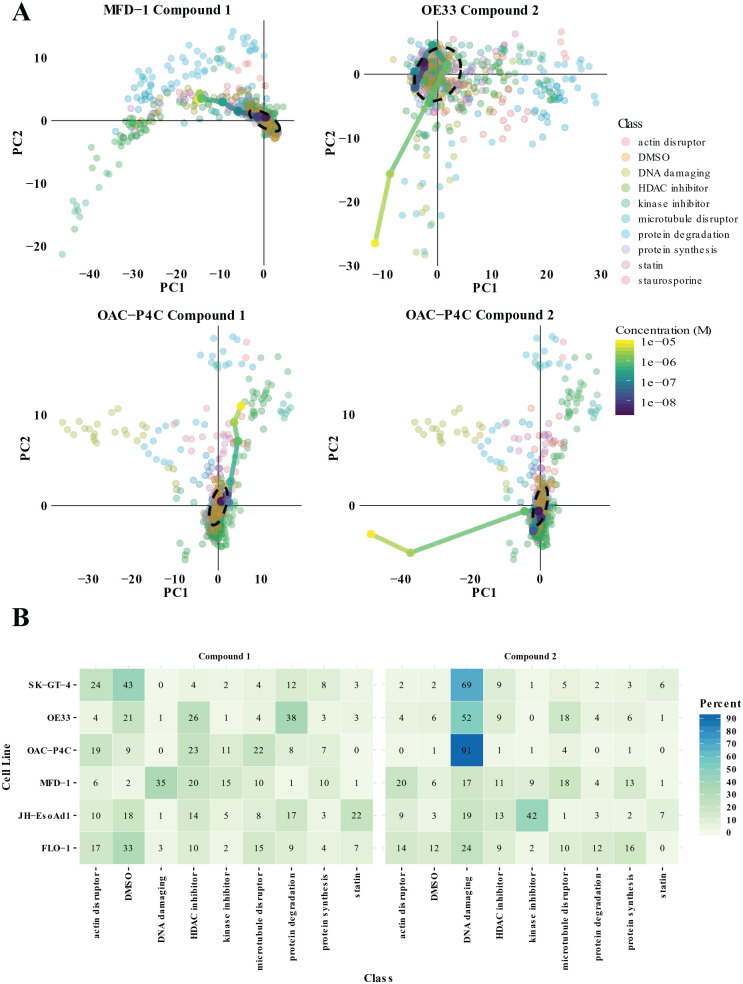
Phenotypic analysis of novel compounds. (**A**) Principal component analysis of compound 1 and compound 2 dose responses overlaid on the reference library for the two most sensitive cell lines for each compound. (**B**) Probabilities expressed as percentages for compound 1 and compound 2 (10 µM) belonging to each class in the reference library for each cell line.

From a subset of 13,000 small-molecule compounds with unknown targets, we further identified a small number of compound hits from our diverse chemical library that showed potent and selective activity for the EAC cell lines. Compound 1 was selective for the OAC-P4C and MFD-1 cells (**Fig. 5A**; **Suppl. Fig. S5**), and machine-learning probabilities for all classes were low (**Fig. 5B**). Compound 2 induced a strong phenotypic dose response in the OAC-P4C and OE33 cell lines only and did not cluster with the reference library of known MoA (**Fig. 5A**; **Suppl. Fig. S6**). Machine learning predicted it to be DNA damaging (91% probability) in the OAC-P4C cells; however, its clustering was distinct, and the machine-learning probability that it is DNA damaging in the OE33 cell line was only 52% (**Fig. 5B**). Therefore. it may in fact represent a novel MoA or be acting to cause DNA damage in a novel way. This indicates that these compounds may be selectively targeting novel esophageal cancer biology. Subsequent transcriptomic and proteomic pathway analysis and target deconvolution studies may reveal the mechanistic pathways involved.

## Discussion

Conventional target-directed drug discovery strategies remain to make any impact on the discovery and translation of effective new treatments for esophageal cancer patients. Key challenges in esophageal cancer include a highly heterogeneous genetic landscape with few mutations in oncogenic drivers, thereby confounding the identification of a clear drug-target hypothesis and modern personalized medicine strategies. In this study, we sought to adapt and evaluate the utility of an advanced high-content phenotypic screening method as an empirical strategy for identifying novel drug targets, MoAs, and pharmacologic classes that target EAC.

Here we have shown that combining high-content screening and image informatics with machine learning can be effective for the identification and mechanistic characterization of hit compounds with selective activity on EAC cell phenotypes. Most multiparametric high-content screening assays and associated machine-learning methods used to predict drug MoA are typically performed on a single cell line. In this study, we have further shown that this format can be applied to heterogeneous panels of cancer cell lines and normal tissue-matched control cells for the identification and prioritization of hit compounds and MoA, which demonstrate selective activity for EAC cells.

Machine learning can be implemented as a tool for multiparametric phenotypic assay quality control (e.g., confirming if the assay is suitable as a discovery platform to classify specific cell phenotypes and elucidate MoA) as well as a tool for MoA deconvolution of hit compounds. Our results demonstrate that this can be standardized across heterogeneous panels of cells.

Following one class of compounds identified in our primary phenotypic screen of 19,555 small molecules tested across all eight esophageal cell lines, we validated antimetabolites as selectively lethal to the EAC lines in vitro following dose-response studies. Using the multiparametric phenotypic information to generate phenotypic dose responses, combined with a reference library of compounds, machine learning, and clustering techniques, we demonstrated the ability to study/predict the MoA of hits from the screen. Here we validated this technique using the antimetabolite hit compounds (methotrexate, pemetrexed, and raltitrexed), showing DNA damage as a likely MoA for the selectivity of these compounds, which is consistent with the literature.^38,39^ These results, together with our identification of hit compounds from our diverse chemical set, which are not classified by our reference set of known MoAs, demonstrates the impact of phenotypic screening in combination with machine learning for MoA studies. This strategy will be used to assess and prioritize novel small-molecule hits from the diverse chemical library screen for further mechanistic studies. From our primary phenotypic screen, we have identified in total 75 compounds that match our hit selection criteria for selective activity across the EAC panel. These 75 hits are an accumulation of the 62 compounds defined by cell morphometric phenotypic analysis and 27 compounds defined by cell proliferation and survival (nuclei count) analysis, with 14 compounds overlapping. The 75 hits shall be further progressed through dose-response studies and secondary assays to confirm and prioritize classes of selective compounds for subsequent drug repurposing and or drug discovery studies.

In addition, using bioinformatic approaches, we hope that integration of phenotypic data with genetic data across our panel of diverse cell lines may provide insight into the selective activity of phenotypic hits and generate the basis for future genetic biomarker–based clinical trials in EAC.

Overall, our high-content EAC assay has proven effective in the identification and mechanistic characterization of hit compounds, demonstrating its utility as a novel empirical strategy for the discovery of new therapeutic targets, chemical starting points, and repurposing of existing drug classes to reignite drug discovery and development in EAC.

## Supplemental Material

Supplemental_Material_for__high_content_phenotypic_profiling_by_Hughes,_et_al – Supplemental material for High-Content Phenotypic Profiling in Esophageal Adenocarcinoma Identifies Selectively Active Pharmacological Classes of Drugs for Repurposing and Chemical Starting Points for Novel Drug Discovery Click here for additional data file. Supplemental material, Supplemental_Material_for__high_content_phenotypic_profiling_by_Hughes,_et_al for High-Content Phenotypic Profiling in Esophageal Adenocarcinoma Identifies Selectively Active Pharmacological Classes of Drugs for Repurposing and Chemical Starting Points for Novel Drug Discovery by Rebecca E. Hughes, Richard J. R. Elliott, Alison F. Munro, Ashraff Makda, J. Robert O’Neill, Ted Hupp and Neil O. Carragher in SLAS Discovery
