# Supplementary material for: High-Content Phenotypic Profiling in Esophageal Adenocarcinoma Identifies Selectively Active Pharmacological Classes of Drugs for Repurposing and Chemical Starting Points for Novel Drug Discovery
Source: SLAS Discov. 2020 May 22;25(7):770–82. doi: 10.1177/2472555220917115 (PMC7372582; doi:10.1177/2472555220917115)
Supplement: Supplemental_Material_for__high_content_phenotypic_profiling_by_Hughes,_et_al – Supplemental material for High-Content Phenotypic Profiling in Esophageal Adenocarcinoma Identifies Selectively Active Pharmacological Classes of Drugs for Repurposing and Chemical Starting Points for Novel Drug Discover [file Supplemental_Material_for__high_content_phenotypic_profiling_by_Hughes,_et_al.pdf]

Supplemental Material

High Content Phenotypic Profiling in Oesophageal Adenocarcinoma Identifies Selectively Active Pharmacological Classes of Drugs for Repurposing and Chemical Starting Points for Novel Drug Discovery

Rebecca E Hughes[1], Richard J R Elliott[1], Alison F Munro[1], Ashraff Makda[1], J Robert O'Neill[2], Ted Hupp[1], Neil O Carragher[1]

[1]MRC Institute of Genetics & Molecular Medicine, The University of Edinburgh, Western General Hospital, Edinburgh, EH4 2XR

[2]Cambridge Oesophagogastric Unit, Cambridge University Hospitals Foundation Trust, Cambridge, CB2 2QQ

Correspondence: Professor Neil Carragher, Cancer Research UK Edinburgh Centre, MRC Institute of Genetics and Molecular Medicine, University of Edinburgh, Edinburgh, EH4 2XR, United Kingdom. Email: n.carragher@ed.ac.uk

Supplementary Table S1. Compound Libraries and screening concentrations.

Library	Concentration (μM)
Prestwick Chemical Library	1
BioAscent 3K Library	10
LOPAC	3
Bespoke Library	1-3
CRUK therapeutics discovery laboratories Library	10-12

Supplementary Table S2. Reference Library of Compounds. 37 compounds and their classes.

Compound	Mechanism of Action
Cytochalasin B	Actin disrupting
Cytochalasin D	Actin disrupting
Latrunculin	Actin disrupting
Camptothecin	DNA damaging
SN38	DNA damaging
Dasatinib	Kinase inhibitor

Saracatinib	Kinase inhibitor
Epothilone B	Microtubule disrupting
Paclitaxel	Microtubule disrupting
Colchicine	Microtubule disrupting
Nocodazole	Microtubule disrupting
Monastrol	Microtubule disrupting
ARQ621	Microtubule disrupting
Barasertib	Microtubule disrupting
ZM447439	Microtubule disrupting
MG132	Protein degradation
Lactacystin	Protein degradation
ALLN	Protein degradation
ALLM	Protein degradation
Cycloheximide	Protein synthesis
Emetine	Protein synthesis
Lovastatin	Statin
Simvastatin	Statin
SAHA	HDAC inhibitor
Panobinostat	HDAC inhibitor
Trichostatin A	HDAC inhibitor
Romidepsin	HDAC inhibitor
Entinostat	HDAC inhibitor
Quisinostat	HDAC inhibitor
Ricolinostat	HDAC inhibitor
Tubastatin A	HDAC inhibitor
Droxinostat	HDAC inhibitor
PCI34051	HDAC inhibitor
TMP195	HDAC inhibitor
LMK235	HDAC inhibitor
CUDC907	HDAC inhibitor
Belinostat	HDAC inhibitor

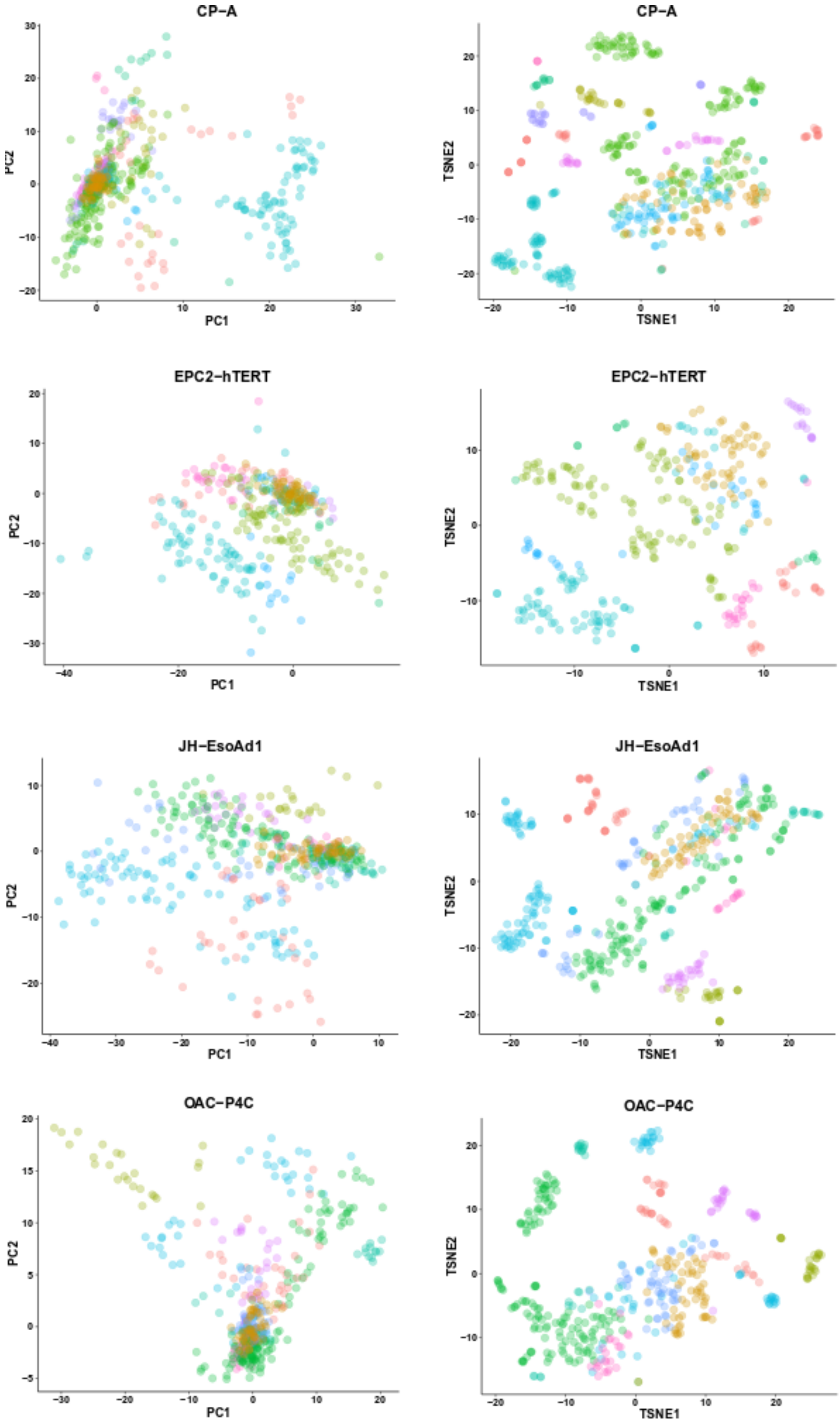
CP−A
PC1
PC2
CP−A
TSNE1
TSNE2
EPC2−hTERT
PC1
PC2
EPC2−hTERT
TSNE1
TSNE2
JH−EsoAd1
PC1
PC2
JH−EsoAd1
TSNE1
TSNE2
OAC−P4C
PC1
PC2
OAC−P4C
TSNE1
TSNE2

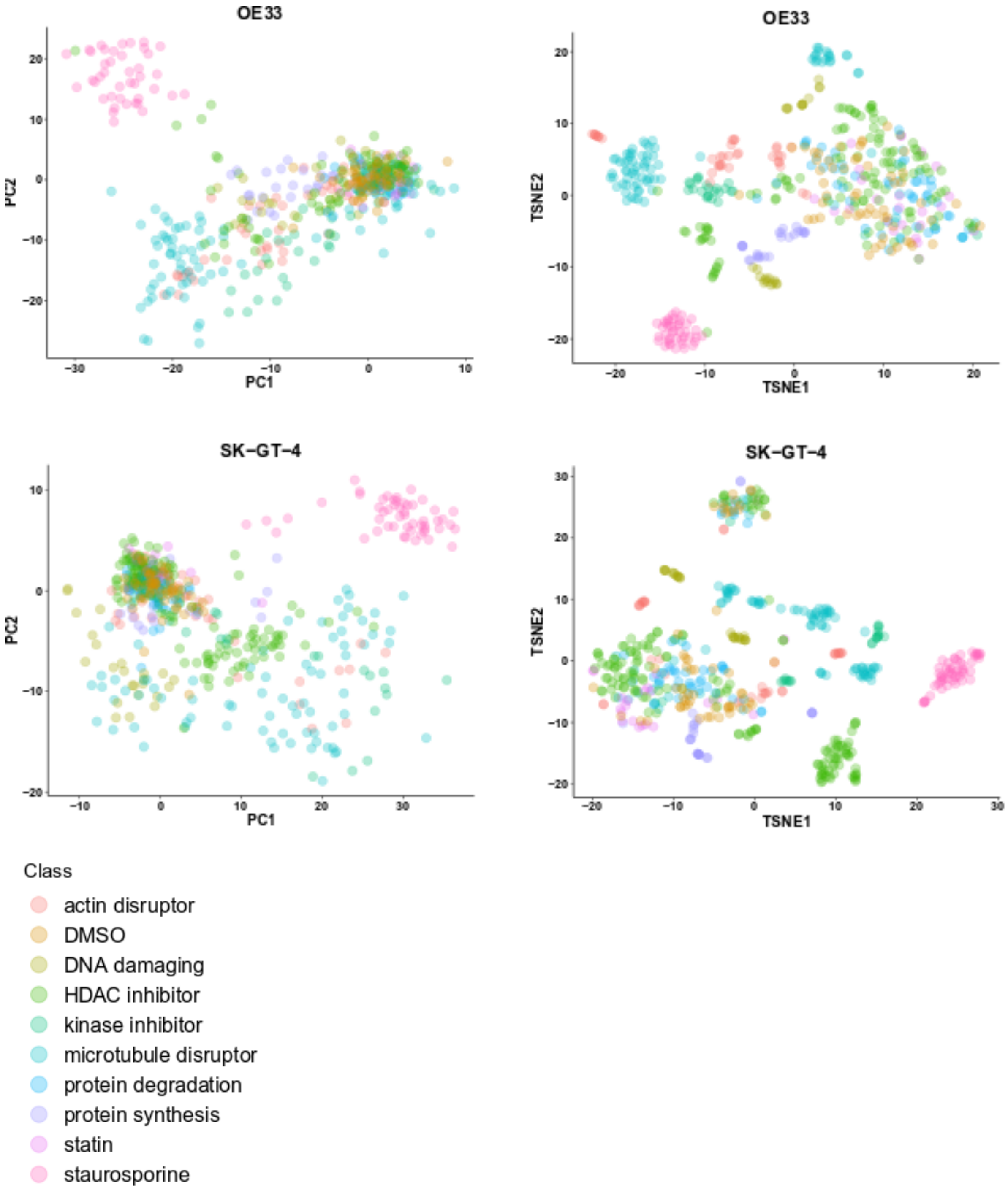

Supplementary Fig. S1. PCA and t-SNE plots. The first two components of principal component analysis (PCA) and t-distributed stochastic neighbour embedding (T-SNE) for the reference library compound treatments for the cell panel (excluding the FLO-1 and MDF-1 cell lines, see Figure 2). Points are coloured by mechanistic class and multiple compound concentrations are plotted.

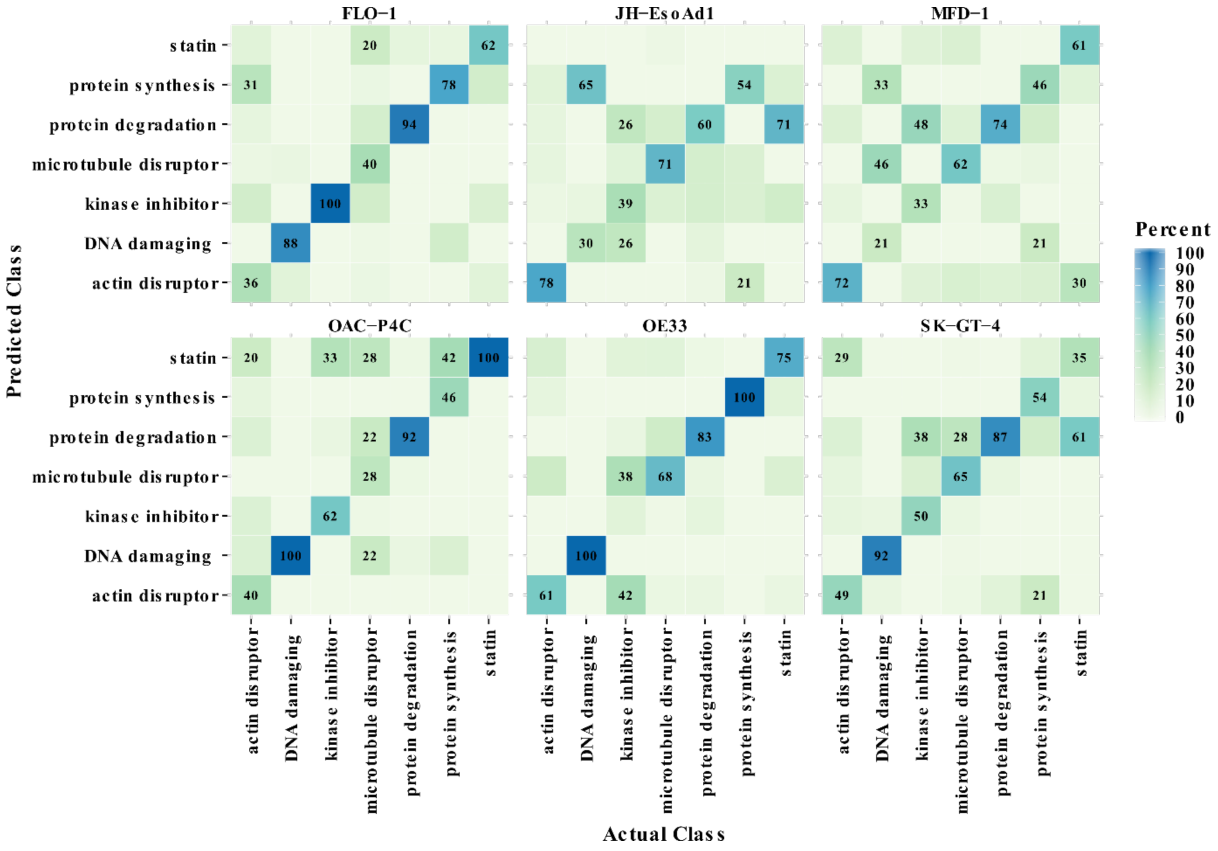

Supplementary Fig. S2. Leave-one-out random forest confusion matrices for reference library of compounds with known mechanisms-of-action. Prediction accuracies for each withheld cell line from a random forest classifier trained on the other five cell lines at a time.

A

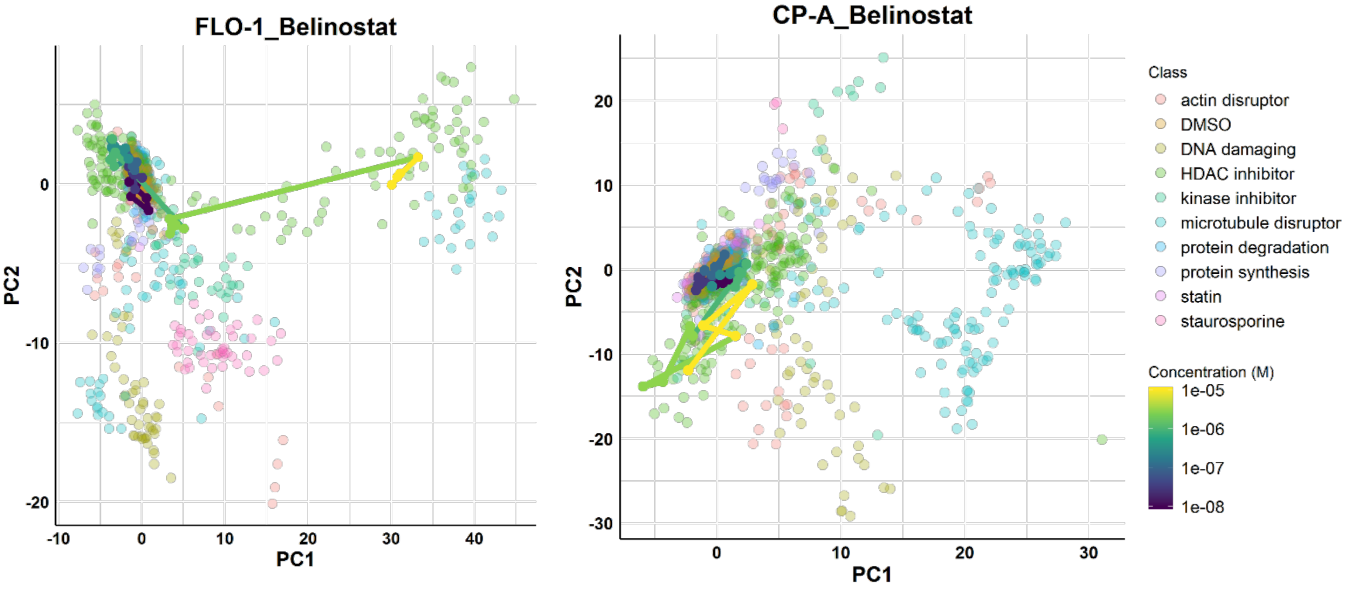

B

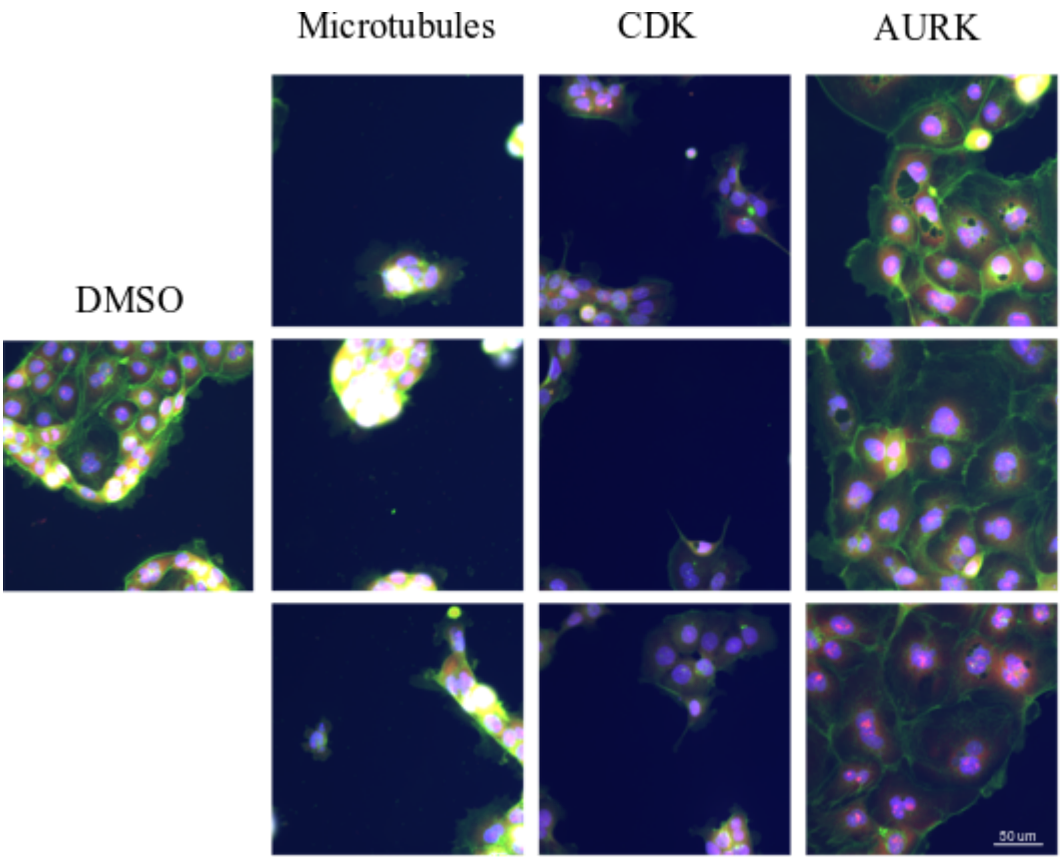

Supplementary Fig. S3. Phenotypic analysis Data. A) Phenotypic dose response for HDAC inhibitor Belinostat. The first two principal components for the feature data from the Belinostat dose response overlaid on reference library for FLO-1 and CP-A cell lines. B) Colour combined images for JH-EsoAD1 cells treated with three compounds from each of three classes; Aurora kinase (AURK) inhibitors, cyclin dependent kinase (CDK) inhibitors, Microtubule disruptors. DAPI channel (blue), TxRED channel (green), Cy3 channel (red). Scale bar is 50 μm.

Supplementary Table S3. Antimetabolite IC50s across the panel of cell lines (nM).

Cell Line	Methotrexate	Pemetrexed	Raltitrexed
JH-EsoAD1	99	110	9
FLO-1	74	224	12
MFD-1	112	263	30
OE33	52	87	4
OAC-P4C	421	10000	31
SK-GT-4	120	870	17

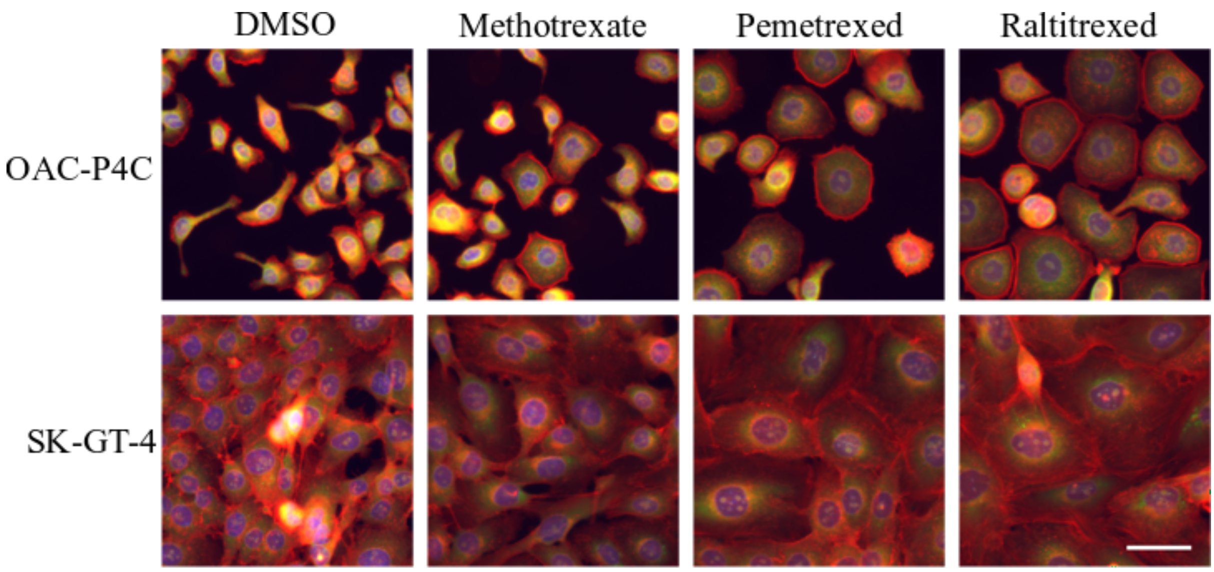

Supplementary Fig. S4. Colour combined images for the antimetabolites Methotrexate, Pemetrexed and Raltitrexed at 10μM in two exemplar cell lines, OAC-P4C and SK-GT-4. Scale bar 50 μm. DAPI channel (blue), TxRED channel (red), FITC channel (Green).

Supplementary Table S4. NanoString normalised counts for Histone H3 subunits. MTX = Methotrexate.

	CPA DMSO	CPA MTX	EPC2 DMSO	EPC2 MTX	FLO1 DMSO	FLO1 MTX	OE33 DMSO	OE33 MTX	SKGT4 DMSO	SKGT4 MTX
HIST1H3H	30,333	29,853	19,088	18,171	18,179	7,925	24,946	9,563	25,135	13,495
HIST1H3G	17,566	17,337	10,793	10,095	17,970	9,243	14,998	5,527	17,573	10,035
HIST1H3B	26,664	25,856	17,285	16,441	28,459	18,330	30,594	15,909	27,481	17,485

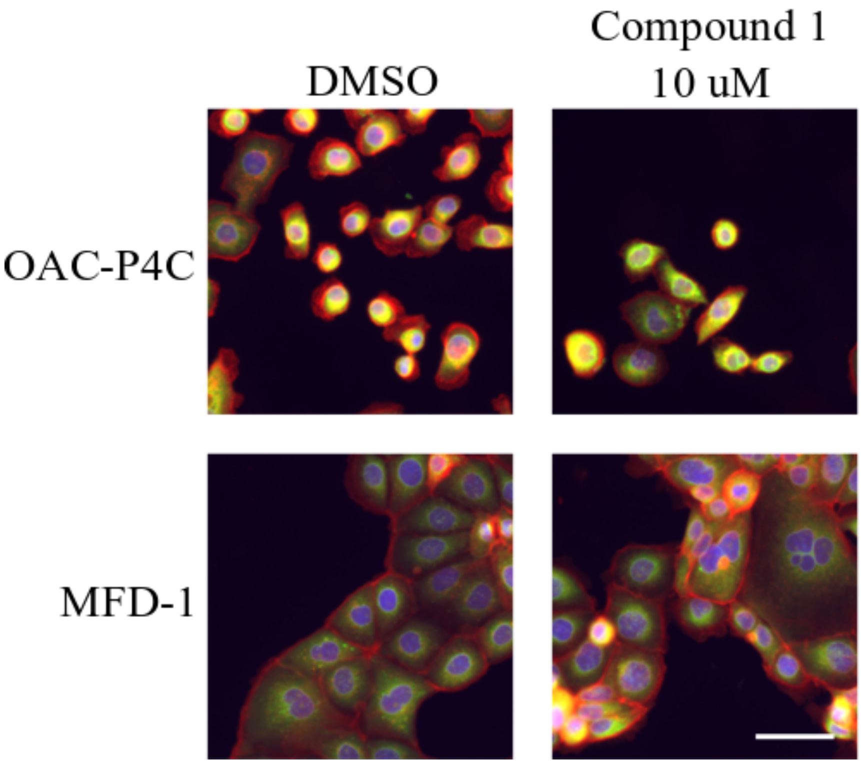

Supplementary Fig. S5. Colour combined images for Compound 1 at 10μM in the two most sensitive cell lines, OAC-P4C and MFD-1. DMSO images included for comparison. Scale bar 50 μm. DAPI channel (blue), TxRED channel (red), FITC channel (Green).

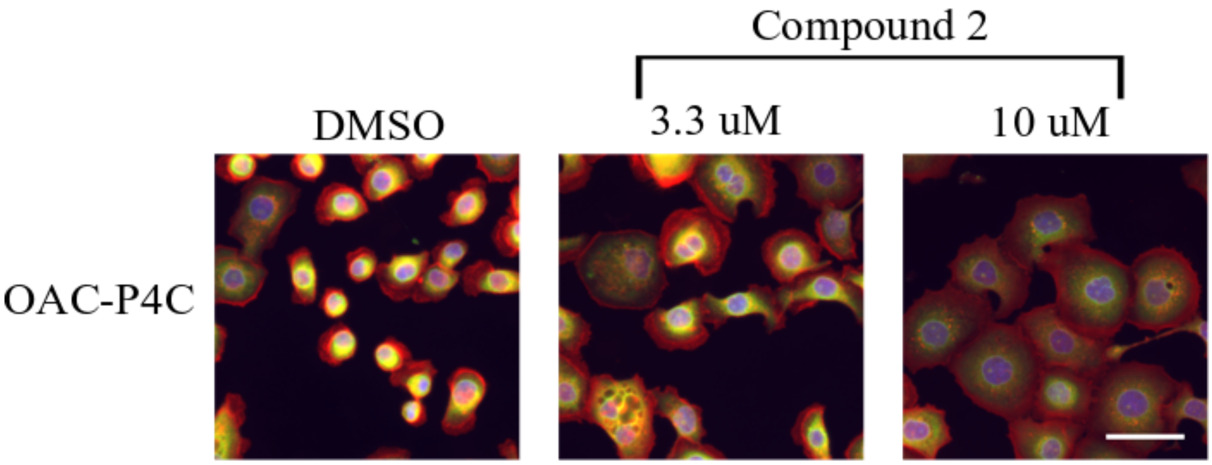

Supplementary Fig. S6. Supplementary Fig. S5. Colour combined images for Compound 2 at 3.3 and 10μM in the most sensitive cell line, OAC-P4C. DMSO image included for comparison. Scale bar 50 μm. DAPI channel (blue), TxRED channel (red), FITC channel (Green).
